# Fluorescent nucleobase analogues for base–base FRET in nucleic acids: synthesis, photophysics and applications

**DOI:** 10.3762/bjoc.14.7

**Published:** 2018-01-10

**Authors:** Mattias Bood, Sangamesh Sarangamath, Moa S Wranne, Morten Grøtli, L Marcus Wilhelmsson

**Affiliations:** 1 Department of Chemistry and Molecular Biology, University of Gothenburg, SE-412 96 Gothenburg, Sweden; 2 Department of Chemistry and Chemical Engineering, Chemistry and Biochemistry, Chalmers University of Technology, SE-412 96 Gothenburg, Sweden

**Keywords:** B-to-Z-DNA transition, fluorescent base analogues, FRET, netropsin, nucleic acid structure and dynamics, quadracyclic adenines, tricyclic cytosines, Z-DNA

## Abstract

Förster resonance energy transfer (FRET) between a donor nucleobase analogue and an acceptor nucleobase analogue, base–base FRET, works as a spectroscopic ruler and protractor. With their firm stacking and ability to replace the natural nucleic acid bases inside the base-stack, base analogue donor and acceptor molecules complement external fluorophores like the Cy-, Alexa- and ATTO-dyes and enable detailed investigations of structure and dynamics of nucleic acid containing systems. The first base–base FRET pair, tC^O^–tC_nitro_, has recently been complemented with among others the adenine analogue FRET pair, qAN1–qA_nitro_, increasing the flexibility of the methodology. Here we present the design, synthesis, photophysical characterization and use of such base analogues. They enable a higher control of the FRET orientation factor, *κ*^2^, have a different distance window of opportunity than external fluorophores, and, thus, have the potential to facilitate better structure resolution. Netropsin DNA binding and the B-to-Z-DNA transition are examples of structure investigations that recently have been performed using base–base FRET and that are described here. Base–base FRET has been around for less than a decade, only in 2017 expanded beyond one FRET pair, and represents a highly promising structure and dynamics methodology for the field of nucleic acids. Here we bring up its advantages as well as disadvantages and touch upon potential future applications.

## Review

### Introduction

The importance of nucleic acid structure and dynamics in the understanding of vital processes in living organisms has led to the development of a large number of techniques for such investigations. Among the most significant ones are NMR [1] and X-ray crystallography [2]. Both techniques offer a high structure resolution and NMR can also provide information on dynamics. However, there are occasions where NMR and X-ray crystallography suffer from drawbacks: the sample amount requirement and biomolecular size restriction for NMR and the difficulties in obtaining crystals and the obvious lack of solution dynamics for X-ray crystallography. An important method for biomolecular structure and dynamics investigations that complements NMR and X-ray, normally at lower resolution, is Förster resonance energy transfer (FRET) [3–4]. FRET and especially single-molecule FRET (as an effect of a low number of biomolecules under study) has the advantage of enabling structure and dynamics investigations in living cells [3,5–6]. FRET is a process that depends on the radiationless energy transfer between a donor and an acceptor molecule [7]. The reason that it can be used as a structure and dynamics technique is that it depends heavily on the distance and relative orientation between the donor and acceptor. Typical distances that can be monitored range between 15–90 Å which well match the dimensions of biomolecules. The efficiency of an energy-transfer process (*E*, between 0 and 100%) can be established using either steady-state or time-resolved fluorescence spectroscopy by comparing fluorescence properties with and without the acceptor molecule present. This efficiency (*E*) depends on the distance (*R*_DA_) between the donor and acceptor as described in Equation 1:

[1]
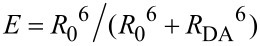


where *R*_0_ is the Förster distance (Equation 2), a characteristic distance of the donor–acceptor pair at which the energy transfer efficiency (*E*) is 50%.

[2]
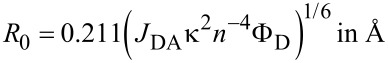


**Figure 1:**
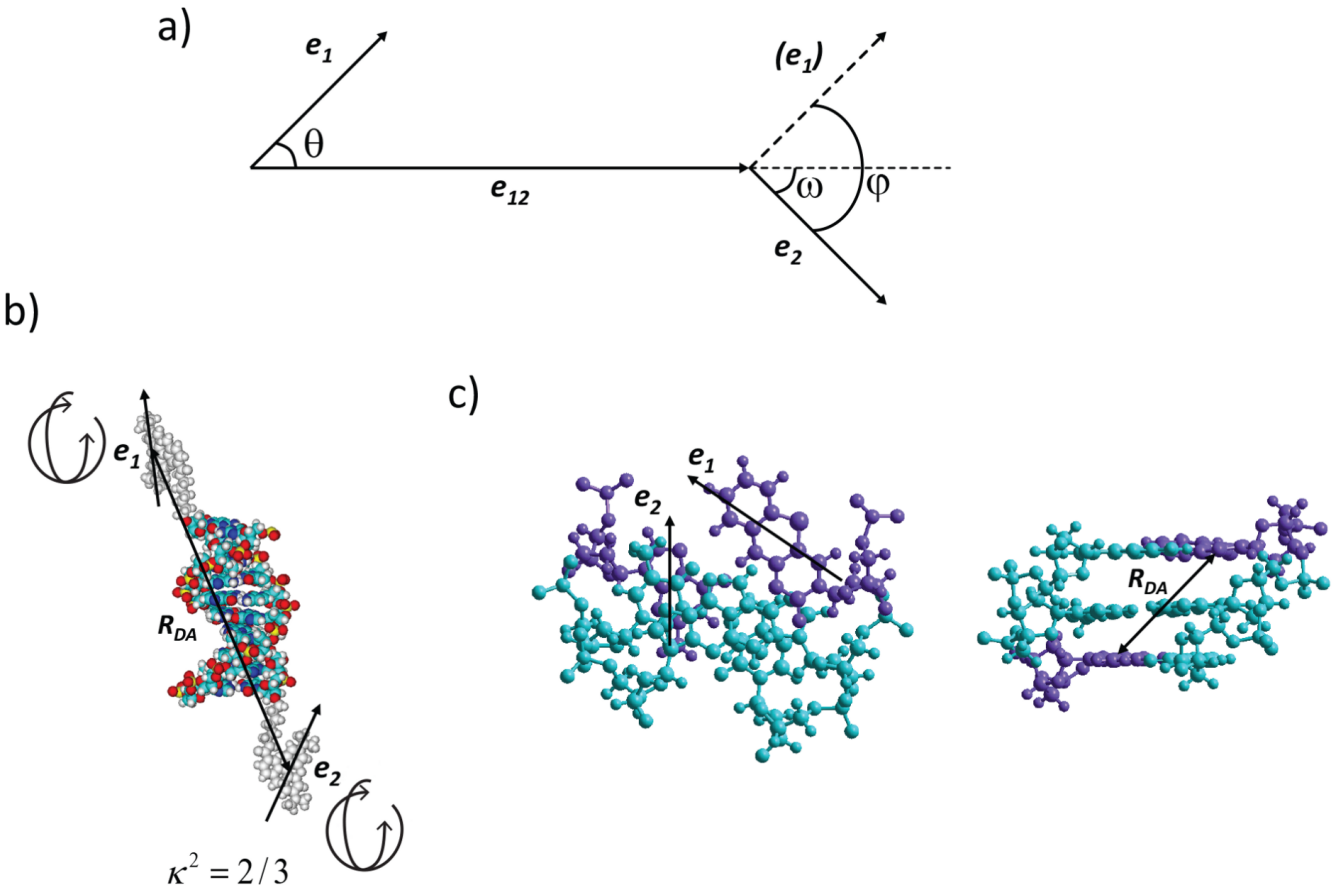
a) Angles and unit vectors used to define the relative orientations of the donor and acceptor transition dipole moments (**e**_1_, **e**_2_) and the separating vector (**e**_12_). b) Illustration of the external fluorophores covalently attached to a DNA and their transition dipole moments (**e**_1_, **e**_2_) with free rotation relative to the DNA, i.e., isotropic orientation, κ^2^ = 2/3. *R*_DA_ is the separation between donor and acceptor. c) DNA top-view (left) and side-view (right) illustrating the typical situation for the virtually static transition dipole moments (**e**_1_, **e**_2_) of fluorescent base analogues in base–base FRET and the distance separating them (*R*_DA_).

As can be seen in Equation 2 the Förster distance depends on the quantum yield of the donor (Φ_D_), the donor/acceptor spectral overlap integral (*J*_DA_, overlap between energies of donor emission and acceptor absorption envelope), the refractive index of the medium (*n*), and importantly the geometric factor (κ, Equation 3). This factor takes the relative orientation of the transition dipole moments of the donor and acceptor into account and, thus, introduces an orientation dependence to *R*_0_ and consequently also to the energy transfer efficiency, *E*. The orientation factor, which ranges between 0 and 4, is governed by Equation 3:

[3]
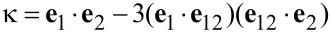


where **e**_1_ and **e**_2_ are the unit vectors of the donor and acceptor transition dipole moments and **e**_12_ the unit vector between their centers (see Figure 1a; κ can also be expressed using the angles in Figure 1a: κ = *cos* φ – 3*cos* θ *cos* ω).

In most FRET applications, an orientation factor κ^2^ of 2/3 is used. This is the correct value for freely rotating, isotropic donor and acceptor molecule orientations (Figure 1b). With an isotropic orientation of the donor and acceptor throughout the experiment the energy transfer efficiency (*E*) is directly related to the distance and the technique becomes a spectroscopic ruler. Such an assumption is often made, both correctly and incorrectly [8–10], for covalently attached external nucleic acid fluorophores like Cy-, Alexa- and ATTO-dyes. This provides a powerful means for measuring long distance ranges (typically 35–90 Å) in nucleic acid-containing systems. However, with the free rotation of the donor and the acceptor the ability of FRET to monitor changes in orientation between them is also lost. With virtually static donor and acceptor molecules (Figure 1c) κ^2^ can be used to improve the structure resolution via the introduction of orientation information, i.e., FRET will also work as a spectroscopic protractor. Several investigations, including the ones by Tor et al. [11], Lewis et al. [12] and Lilley et al. [13], have taken significant steps in the direction of introducing orientational information into nucleic acid FRET. Recently, our group took this progress one step further and introduced base–base FRET [14], where the donor and acceptor molecules are nucleobase analogues [15–16]. With the donor and acceptor molecules rigidly stacked in the base-stack of the nucleic acid (Figure 1c) this approach provides highly accurate orientation information and has the potential to increase the structure and dynamics information obtained in a nucleic acid FRET experiment. Later Asanuma et al. introduced base-stacked aromatic moieties [17], not working as nucleobase analogues, which also can be used to provide information about orientation.

In this review we will focus on the FRET between fluorescent base analogues, i.e., base–base FRET, the theory behind it, the increased accuracy in orientation factor κ^2^ as an effect of their position inside the base-stack, other advantages and disadvantages compared to FRET in nucleic acids using external fluorophores like Cy-, Alexa- and ATTO-dyes as well as finally summarize some of its recent applications. The field started less than a decade ago with the introduction of the first fluorescent nucleobase analogue FRET pair, tC^O^–tC_nitro_, and we divide this review into three parts: the first one dealing with the synthesis of the key players of base–base FRET, i.e., the base analogue donor and acceptor molecules, the second one dealing with their photophysical properties and the third one dealing with their application in studying nucleic acid-containing systems.

### Synthesis of fluorescent base analogues

**Figure 2:**
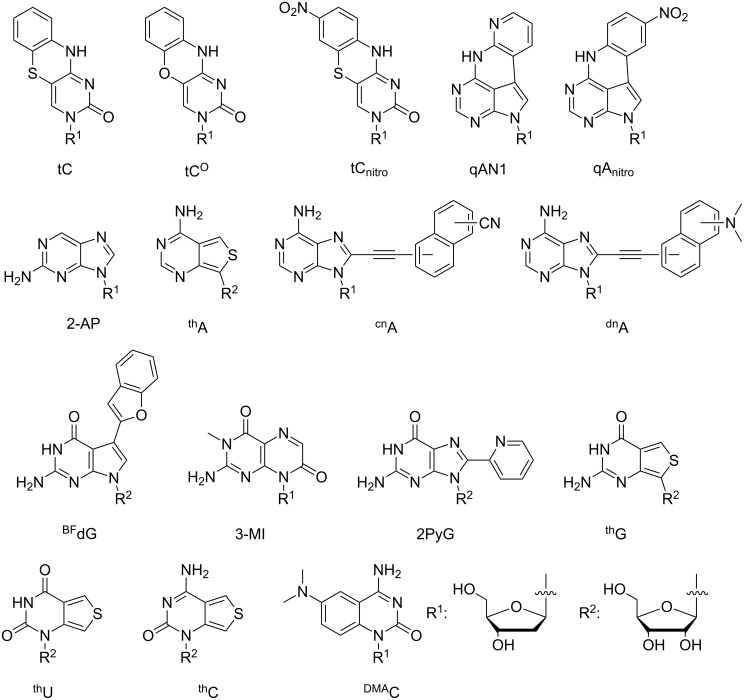
Notable recent examples of fluorescent base analogues. For ^cn^A and ^dn^A the attachment point to the substituted naphthalene moiety has been varied.

The development of synthesis methods of nucleobase analogues remains a challenge. This is mainly due to the presence of multiple reactive functional groups both on the nucleobase as well as the sugar moiety and requires the introduction of orthogonal protection groups. A careful consideration of protection groups is paramount as an extensive use adds additional steps as well as complexity to the synthesis. The design and synthesis of fluorescent nucleobase analogues (FBAs) add on additional challenges such as obtaining features that introduce useful photophysical properties, for example, extended conjugation. As an effect of the need for hydrogen bonding properties, size restriction and sterical effects these demands are often conflicting [15,18–19]. However, there is an increasing number of exceptions to this and since the pioneering work of Ward et al. on adenine analogues [20] a whole range of small modifications to nucleobases, such as the 8-vinyldeoxyadenosine [21], has led to the introduction of fluorescence. Considering the differences in the structures of purines and pyrimidines, adenine is unique amongst the natural bases as it offers several sites for modifications: C2, C8, the C6 exocyclic amino functionality and the vastly explored N7 to C7 substitution leading to 7-deazaadenines. On the contrary, for guanine only the C8 and the C2 exocyclic amine are directly accessible for modifications as well as the previously mentioned 7-deaza substitution. Looking at the monocyclic pyrimidines, only the C5 and C6 positions are available for modifications without directly perturbing the base-pairing properties. The subtle differences between the nucleobases within a class could lead one to believe that the chemistry developed for modifications of adenine would translate easily to guanine. Unfortunately, the variety of functional groups requires different protection group strategies and, moreover, changes the reactivity of the nucleobase. Since the discovery of the gold standard of fluorescent base analogues, 2-AP, a multitude of adenine FBAs has emerged [22]. Notable recent examples of adenine FBAs (see Figure 2 for chemical structures) include C8 to S8 thio-RNA analogue ^th^A [23], the C8-naphtalene substituted adenines ^cn^A and ^dn^A [24], as well as our own quadracyclic qAN1 [25]. A handful of fluorescent guanine analogues has been synthesized and characterized and includes the recent turn-on probe BFdG, 3-MI, 2PyG, as well as the emissive RNA analogue ^th^G [23,26–28]. Some notable pyrimidine analogues include our tricyclic analogues tC and tC^O^ [29–31], pyrrolo-dC [32] and its derivatives [33] as well as ^th^U, ^th^C [23] and ^DMA^C [34]. Apart from tC, tC^O^, qAN1 and ^th^G, FBAs have not yet been utilized in base–base FRET applications. However, the brightest of these FBAs combined with a matching donor or acceptor molecule could potentially also be used in base–base FRET in the future.

### Synthesis of cytosine analogues for base–base FRET in DNA

**Scheme 1:**
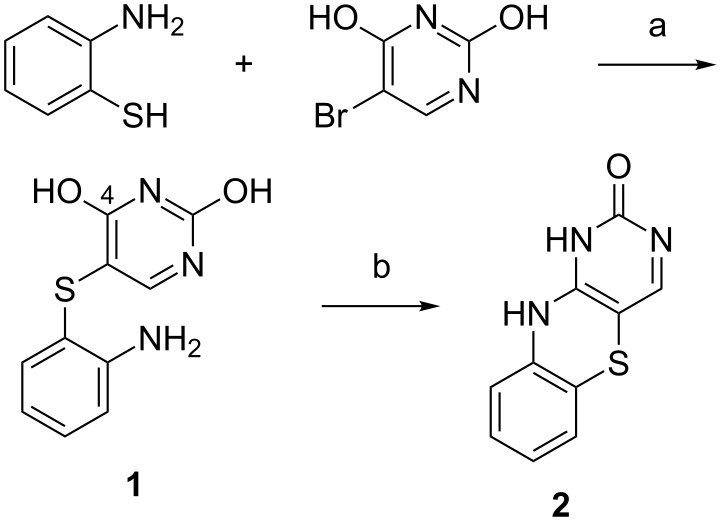
Synthesis of the tricyclic cytosine aromatic core [39]. (a) Ethylene glycol, K_2_CO_3_, 120 °C, 1 h, 40%; (b) EtOH, 1 M HCl, reflux, 16 h, 75%.

We have put considerable effort into developing the family of fluorescent base analogues known as the tricyclic cytosines (tC) [14,29–31,35–38]. The aromatic core of tC was first prepared by Roth et al. in 1963 as part of a study to obtain pharmacologically active compounds structurally similar to phenothiazines [39]. Compound **1** (Scheme 1) was readily prepared from condensation of 2,4-dihydroxy-5-bromopyrimidine with 2-aminothiophenol under basic conditions at elevated temperatures and was obtained in 40% isolated yield [40]. Ring-closing of compound **1** to obtain compound **2** was achieved by an acid-catalyzed cyclization which was found to be general for a large set of 4-hydroxy-5-(*o*-aminoarylthio)pyrimidines [39]. The mechanism was thought to proceed via protonation of a pyrimidine ring nitrogen which activates it to nucleophilic attack by an unprotonated anilino nitrogen on the positive C4 of the pyrimidine ring which carries the hydroxy group. The formed complex eliminates water and yielded the cyclization product in 75% after isolation.

**Scheme 2:**
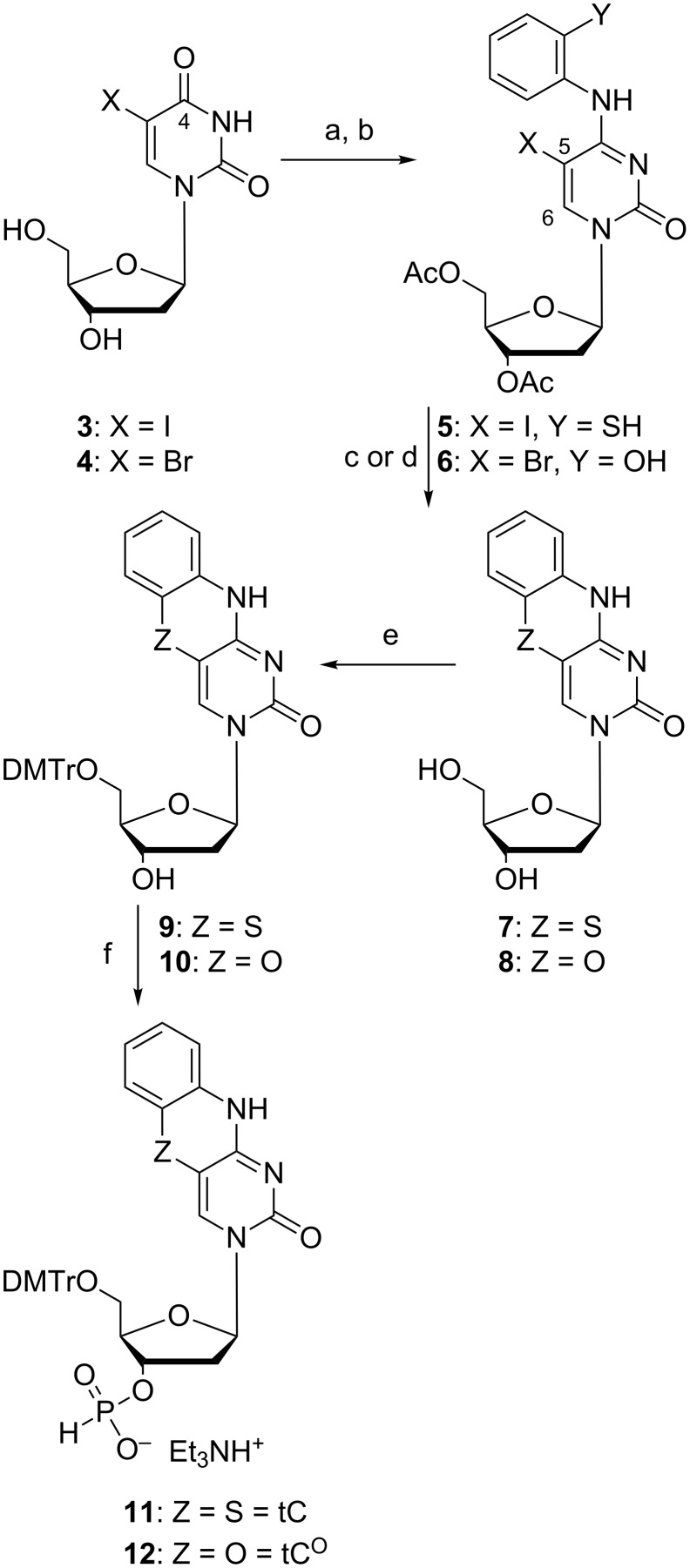
Synthesis of protected tC and tC^O^ deoxyribose phosphonates [41]. (a) Ac_2_O, pyridine, rt; (b) 2-mesitylenesulfonyl chloride, TEA, then 2-aminothiophenol or 2-aminophenol, DBU, rt, 27% and 54% yield, respectively, over two steps; (c) *t*-BuOK in EtOH, reflux, 38%; (d) NH_3_ in MeOH, rt, then 10 equiv of KF, EtOH, reflux; (e) 4,4´-dimethoxytrityl chloride, pyridine, rt, yielding 50% of compound **10** over three steps; (f) 2-chloro-4*H*-1,3,2,-benzodioxaphosphorin-4-one, pyridine, DCM, 0 °C, 71% over two steps and 80%, respectively.

In an effort to increase the thermodynamic stability of oligonucleotide duplexes for antisense purposes, Lin et al. turned to size-expanded nucleobase analogues [41]. To this end they wanted to use the aromatic ring system previously developed by Roth et al. [39] as a nucleobase analogue to furnish greater π–π interactions with the natural bases and possibly also to increased hydrophobic effects. A new strategy for the preparation of tC analogues was used, starting from 5-iodo or 5-bromo-2´-deoxyuridine (Scheme 2) [41]. Compounds **3** and **4** were reacted with acetic anhydride in pyridine to protect the deoxyribose hydroxy groups. The O4 position was then activated by sulfonylation using 2-mesitylenesulfonyl chloride. The subsequent condensation with the appropriate 2-aminothiophenol or 2-aminophenol afforded compounds **5** and **6**, respectively. Refluxing **5** with *t*-BuOK in EtOH generated **7** in 38% isolated yield. When **6** was treated with the same cyclization conditions as **5** only dehalogenation was observed. Compound **8** was obtained by first removing the acetyl protecting groups using ammonia in MeOH, followed by cyclization by refluxing deprotected **8** with KF in EtOH. Presumably, a transient Michael addition of the hydroxy group to the C6 position of compound **6** increases the reactivity of the C5 position towards substitution. Standard dimethoxytritylation of compounds **7** and **8** furnished product **9**, which was used in the next step without isolation and **10** in 50% yield over three steps, respectively. Lastly, phosphitylation yielded the corresponding H-phosphonates **11** in 71% yield over two steps and **12** in 80% yield (Scheme 2).

**Scheme 3:**
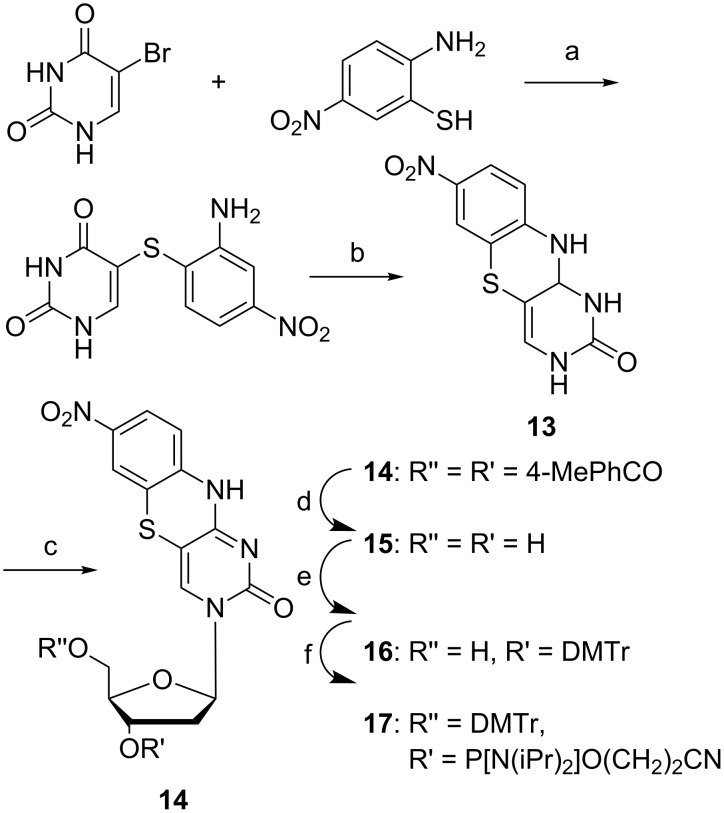
Synthesis of protected tC_nitro_ deoxyribose phosphoramidite [14]. a) aq NaOH, 24 h, reflux; b) EtOH, HCl, 24 h, reflux, 15% over two steps; c) DMF, toluene, 3,5-di-O-*p*-toluoyl-α-D-erythro-pentofuranosyl, NaH, 18 h, rt, 11%; d) MeONa, MeOH, 18 h, rt, 71%; e) pyridine, DMAP, DMTr-Cl, 18 h, rt, 74%; f) DCM, DIPEA, 2-cyanoethyl-*N*,*N*-diisopropylchlorophosphoramidite, 1 h, rt, 93%.

In 2001, tC was reported as a fluorescent nucleobase analogue [39]. The tricyclic core was synthesized as reported by Roth et al., and subsequently functionalized with a carboxylic acid handle for PNA labeling [39]. In 2003, tC [35] was synthesized bearing a 2´-deoxyribose functionality and thoroughly photophysically characterized (vide infra). tC was later functionalized with a phosphoramidite and incorporated into oligonucleotides [30]. However, the fully detailed synthesis with complete characterization was published in 2007 as a Nature Protocol paper [37]. The aromatic core of tC was prepared according to the procedure of Roth et al. (Scheme 1), followed by a glycosylation using the sodium-salt method as later also performed in the synthesis of tC_nitro_ in 2009 (reaction c, Scheme 3) [14,42]. The synthesis was finished by standard DMTr protection and phosphitylation furnishing tC deoxyribose phosphoramidite in a total of 2.1% yield over 6 steps [43–44]. In 2008, the oxo-analogue tC^O^, which Lin et al. initially prepared in 1995 [41], was re-synthesized in order to characterize its photophysical properties, using the same procedure except that *p*-toluoyl protecting groups rather than acetyl were used [31].

In 2009, we published the first base–base FRET system using tC^O^ and tC_nitro_ [14]. Nitro groups introduce an increased charge-transfer character to chromophores, which generally results in absorption at lower energies [38,45]. Hence, tC_nitro_ was envisioned to be able to accept the energy transferred from tC or tC^O^, and, thus serve as a FRET acceptor. The synthesis of tC_nitro_ followed the procedure of Roth et al. [39] to furnish the aromatic core **13** (Scheme 3). Compound **13** was then glycosylated by making the sodium salt and reacting it with Hoffer´s α-chloro sugar yielding **14** in 11% yield after isolation [46]. The *p*-toluoyl protection groups were cleaved by sodium methoxide in MeOH, which yielded the free nucleoside **15** in 71%. Standard DMTr protection furnished compound **16** which was then activated for oligonucleotide solid-phase synthesis (SPS) by phosphitylation using CEP-Cl. The total yield of tC_nitro_ deoxyribose phosphoramidite was 0.8% over 6 steps where the acid-catalyzed cyclization as well as the glycosylation proved challenging. The latter two steps proceeded with a yield of 15% or less (**17**, Scheme 3).

**Scheme 4:**
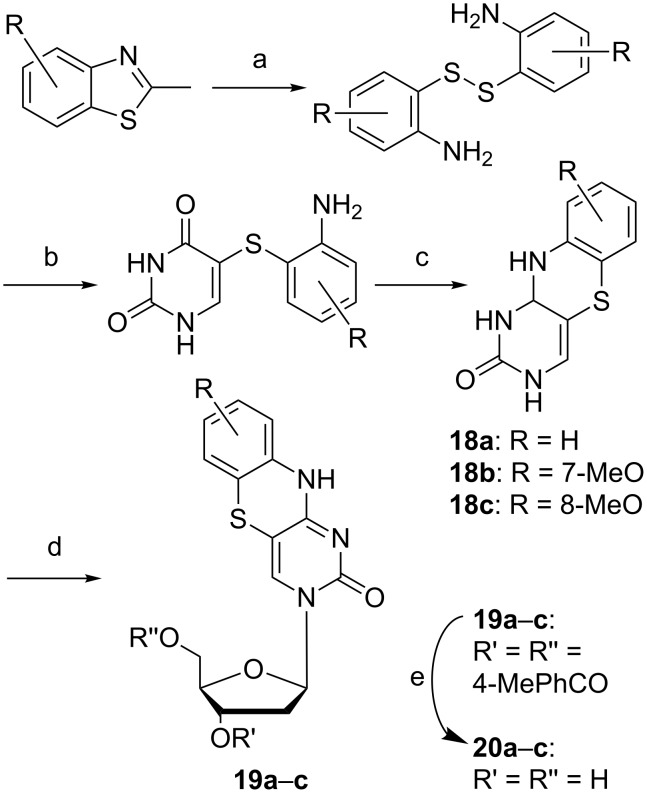
Improved synthesis of tC and tC derivatives, where R = H, 7-MeO or 8-MeO [47]. a) H_2_NNH_2_ followed by H_2_O_2_, 20 h, 100 °C, 60–98%; b) PEt_3_, H_2_O, diglyme, then Na_2_CO_3_ and 5-bromouracil, rt to 120 °C, 2 h, 24–86%; c) HCl, butanol, 120 °C, 24–72 h, 27–86%; d) BSA (bis(trimethylsilyl)acetamide), Hoeffer´s α-chloro sugar, SnCl_4_, 0 °C to rt, 2 h, 12–41%; e) NaOMe, MeOH, 30 min, 69–90%.

A new synthetic approach to access substituted tricyclic cytosines was envisioned in 2014 by Rodgers et al. (Scheme 4). This protocol increased the yield of the parent compound tC from 10% up to 43% in the glycosylation step of the previously prepared tC nucleobase (Scheme 4) [47]. This was achieved by activation of the aromatic core of compounds **18a**–**c** via trimethylsilylation using BSA (bis(trimethylsilyl)acetamide) [47], instead of the sodium-salt method [42]. The deoxyribosylation was then achieved using the same Hoffer’s α-chloro sugar, but in presence of a Lewis acid yielding the protected nucleosides **19a**–**c** [48]. The cleavage of the protection groups was achieved with sodium methoxide to furnish compounds **20a**–**c**.

**Scheme 5:**
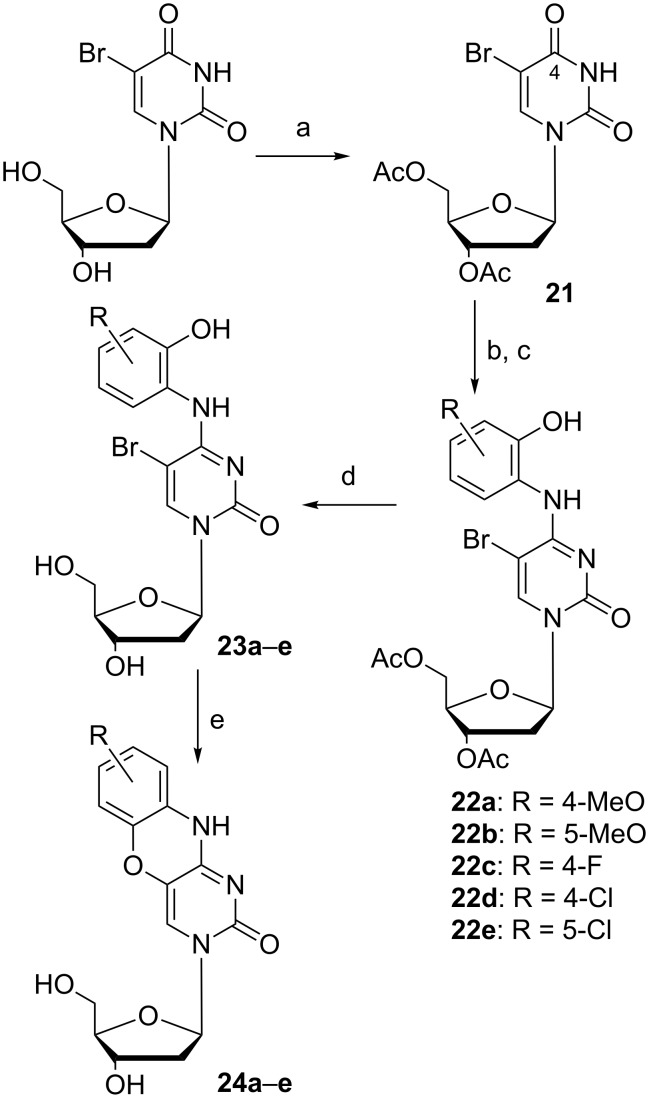
Improved synthesis of tC^O^ derivatives [47]. a) Ac_2_O, pyridine, 16 h, rt, 85%; b) PPh_3_, CCl_4_, DCM, 5 h, 44 °C; c) DBU, DCM, R = 4-MeO, 5-MeO, 4-F, 4-Cl, 5-Cl, 15 min, 0 °C; d) NaOMe, MeOH, 3–4 h, rt, 40%, 48%, 61%, 20%, and 41%, respectively, over three steps; e) KF, 18-crown-6, diglyme, 1–2 h, 120 °C, 20% for R = 8-MeO, 11% for R = 7-MeO, 11% for R = 7-F, 24% for R = 8-Cl, and 3% for R = 7-Cl.

The improved synthetic route to tC^O^ derivatives started from the same 3´,5´-di-*O*-acetyl-5-bromo-2´-deoxyuridine (**21**, Scheme 5) as Lin et al. used, but was instead activated for a condensation using Appel chemistry [41,49]. Compound **21** was activated using PPh_3_ in CCl_4_ which converts the O4 to a 4-Cl and used in situ with various substituted 2-aminophenols in the presence of the strong base DBU which resulted in compounds **22a–e**. A subsequent protection group removal yielded compounds **23a–e** and made the scaffold ready for cyclization. Initially, CsF was used in place of KF, however, the hygroscopic nature of CsF made it impractical to handle. Instead, KF was used in combination with 18-crown-6 in anhydrous diglyme which furnished compounds **24a–e** in modest 3–24% yields after isolation (Scheme 5) [47].

**Scheme 6:**
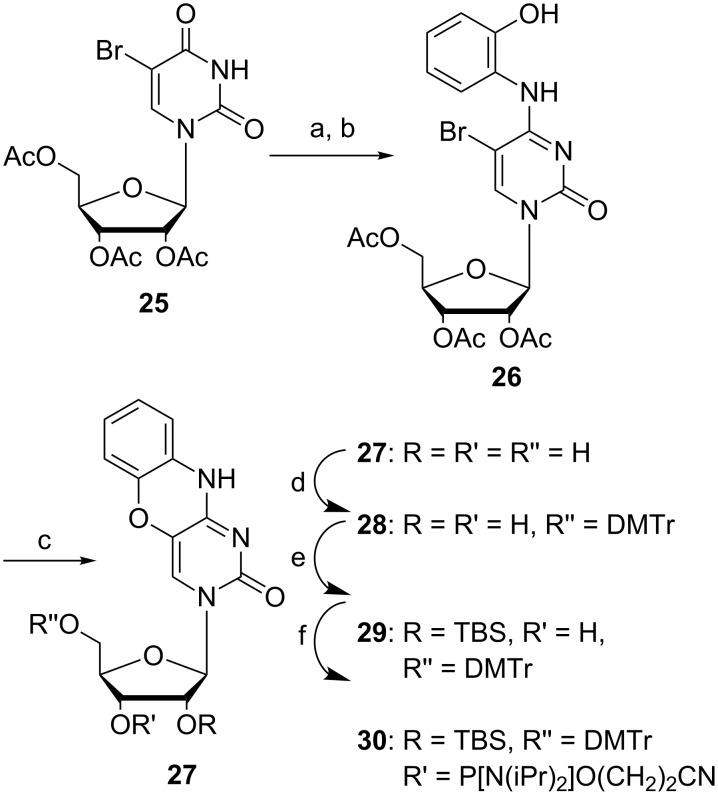
Synthesis of protected tC^O^ ribose phosphoramidite [50]. a) MesSO_2_Cl, DIPEA, MeCN, 4 h, rt; b) 2-aminophenol, 30 min, rt, 71% over two steps; c) KF, EtOH, 2 h, MW 140 °C, 86%; d) DMTr-Cl, pyridine, 1.5 h, rt, 72%; e) AgNO_3_, TBDMS-Cl, pyridine, THF, 4 h, rt, 76%; f) 2-cyanoethyl-*N*,*N*-diisopropylchlorophosphoramidite, DIPEA, THF, 1 h, rt, 93%.

Recently, our group gained interest in RNA chemistry and therefore revisited the synthesis of tC^O^ containing a ribose unit instead of a deoxyribose [50]. By simply activating the O4 of **25** (Scheme 6) using 2-mesitylenesulfonyl chloride and DIPEA in MeCN, the successful condensation with 2-aminophenol was achieved and afforded compound **26** in 71% yield. The cyclization of **26**, which previously suffered from low yields, was effectively obtained in 86% yield by using an excess of KF in ethanol and microwave heating at 140 °C. Conveniently, at the same time all the three acetyl protecting groups were cleaved and the free nucleobase was isolated via precipitation. A 5´-DMTr protection followed by 2´-TBDMS protection and phosphitylation using CEP-Cl generated the fully protected monomer ready for solid-phase synthesis [50]. The complete synthesis of the RNA building block of tC^O^ was in this way achieved over five steps with a total yield of 28%, improved from the four step DNA building block synthesis of tC^O^ by Lin et al. of 22% [41].

### Synthesis of adenine analogues for base–base FRET

**Scheme 7:**
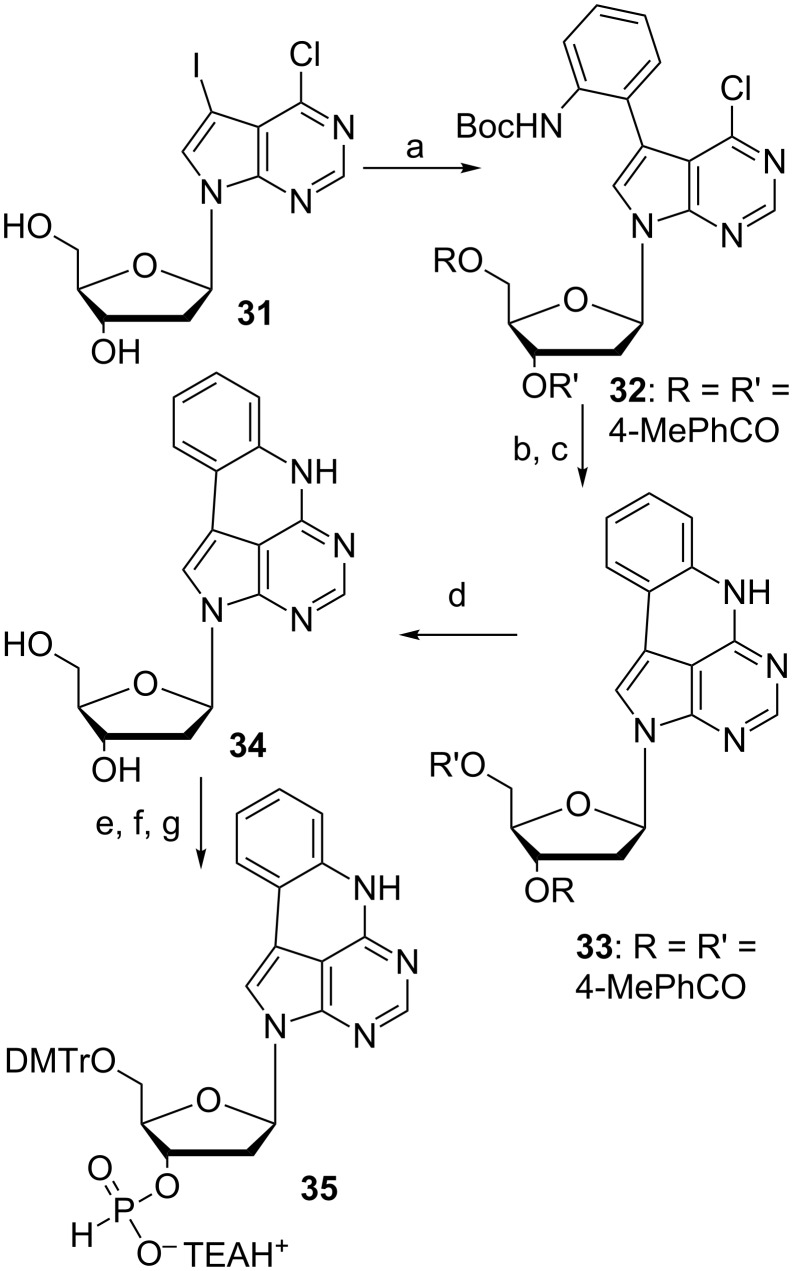
Synthesis of protected deoxyribose qA [51]. a) *N*-(*tert*-Butoxycarbonyl)-2-(trimethylstannyl)aniline, (Ph_3_P)_2_PdCl_2_, DMF, 24 h, 60 °C, 68%; b) DABCO, DBU, DMF, 21 h, 75 °C; c) 25% TFA in CH_2_Cl_2_, 3 h, rt, 96% over two steps; d) NaOMe, MeOH, rt, 64%; e) DMTr-Cl, pyridine, 65%; f) 2-chloro-4*H*-1,3,2-benzodioxaphosphorin-4-one, pyridine, CH_2_Cl_2_, 30 min, 0 °C to rt; g) aq triethylammonium bicarbonate, 52% over two steps.

Buhr et al. were interested in developing modified adenosines that could thermodynamically stabilize double-stranded nucleic acids [51]. In 1999, a short synthesis article regarding quadracyclic adenine, qA, was published, however, it lacked a full experimental procedure (Scheme 7). The synthesis started from 6-chloro-7-iodo-7-deazapurine functionalized at the N-9 position with Hoffer’s α-chloro sugar (**31**, Scheme 7). This material was functionalized further using a Stille coupling to afford a mono-Boc-protected *o*-iodoaniline **32** in 68% yield after isolation [52]. The cyclization was performed via nucleophilic aromatic substitution with DBU and DABCO. Presumably DABCO activates the chlorine and modifies it into a better leaving group allowing the sterically hindered base DBU to abstract a proton from the protected aniline which allows the cyclization. Standard Boc deprotection using TFA gave compound **33** in 96% over two steps. This was followed by *p*-toluoyl deprotection using sodium methoxide in methanol to afford **34** in 64% yield after isolation. Then, the material was protected with DMTr-Cl yielding the protected nucleoside in 65% yield. Subsequent phosphitylation followed by salt-formation finally furnished compound **35** in 52% over two steps.

**Scheme 8:**
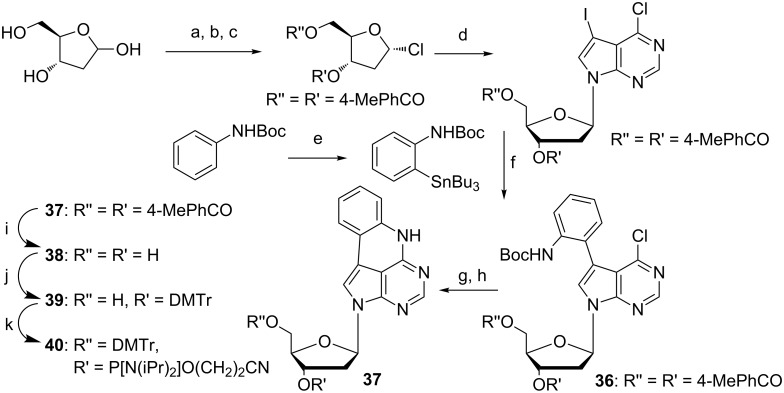
Synthesis of protected deoxyribose qA for DNA SPS [53]. a) AcCl, MeOH, rt, 40 min; b) *p*-toluoyl chloride, pyridine, overnight, 0 °C to rt; c) AcCl, AcOH, H_2_O, 0 °C, 36% over three steps; d) NaH, MeCN, 30 min, rt, then 2 h at 60 °C, 73%; e) *t*-BuLi, SnBuCl_3_, THF, 2h, −78 °C, 65%; f) Pd(PPh_3_)_4_, CuI, CsF, DMF, 1 h, 100 °C, 55%; g) DABCO, DBU, DMF, 16 h, 75 °C; h) 25% TFA in CH_2_Cl_2_, 1.5 h, 0 °C to rt, 46% over two steps; i) NaOMe, MeOH, overnight, rt, 61%; j) DMTr-Cl, pyridine, 1 h, rt, 68%; k) 2-cyanoethyl-*N*,*N*-diisopropylchlorophosphoramidite, DIPEA, CH_2_Cl_2_, 1.5 h, rt, 79%.

Since the quadracyclic adenine presented an overall structural similarity with adenine and keeping a very rigid heterocyclic system suggesting few options for the molecule to decay from excited states via non-radiative processes, in 2012 we decided to re-synthesize the quadracyclic adenine according to the procedure of Buhr et al. (Scheme 8) [51,53]. However, in our hands the vital cyclization reaction starting from compound **36** (Scheme 8) never provided more than a 46% yield of **37** after TFA deprotection of the Boc group compared to the previously reported 96% [51].

**Scheme 9:**
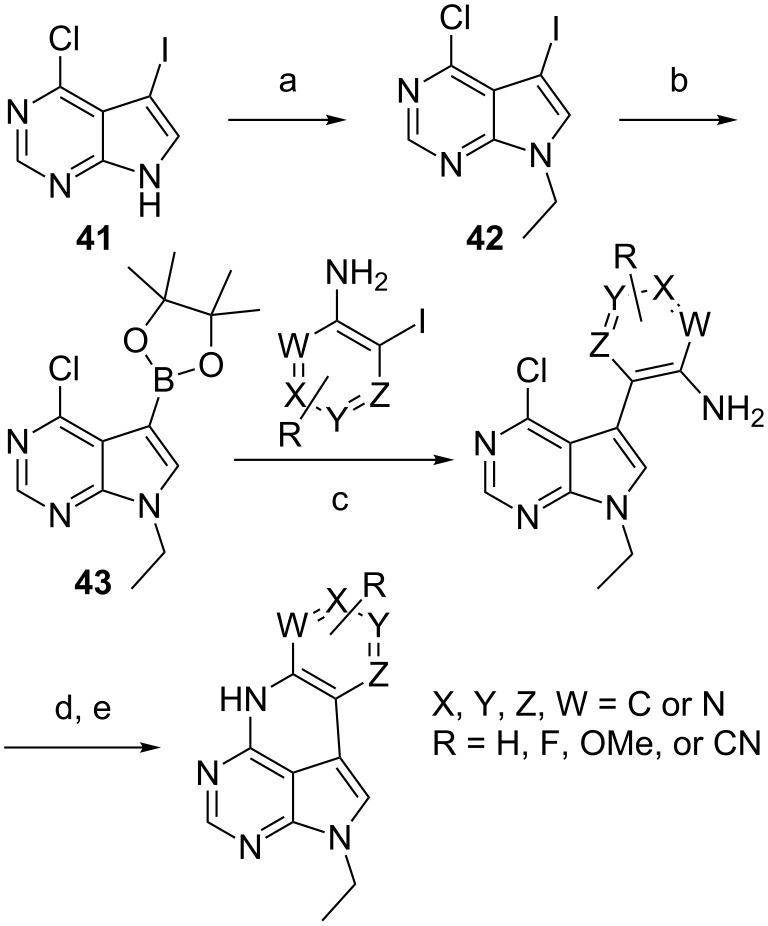
Synthesis of qA derivatives. a) EtI, Cs_2_CO_3_, DMF, 4 h, rt, 90%; b) HBPin, Pd(PPh_3_)_4_, Et_3_N, 1,4-dioxane, 24 h, 80 °C, 86%; c) PdCl_2_(PPh_3_)_2_, K_3_PO_4_, MeCN, H_2_O, 80 °C, 2 h, 56–86%; d) TMS-Cl, THF, 30 min, rt; e) LiHMDS, THF, 100 °C, 3 h, 33–71%.

The base-pairing properties of qA with T and selectivity were found to be excellent. Moreover, the melting temperature of the oligonucleotides remained close to those of unmodified sequences indicating that qA is an excellent adenine analogue [53]. Unfortunately, the photophysical properties of qA were not satisfactory for an internal FRET fluorophore and, thus, we moved on by modifying the quadracyclic aromatic core but leaving the advantageous base-pairing properties. To this end, we needed to develop a more straightforward and versatile synthetic route. The Stille coupling was changed to a Suzuki–Miyaura coupling and the cyclization was performed directly starting from the free aniline nitrogen, as we found that Boc protection was required only for cyclization when using DBU and DABCO. To faster screen a larger set of new compounds for fluorescent properties we envisioned that it was unnecessary to carry the entire sugar moiety through the synthesis. Thus, by alkylation of 6-chloro-7-iodo-7-deazapurine (**41**, Scheme 9) followed by a Miyaura-style borylation of compound **42**, inspired by Thompson et al. we achieved compound **43** in a yield of 77% over two steps [54]. This material was functionalized in two different studies: first by using pyridine-type anilines and later with R-group modifications to the top ring (Scheme 9) [55–56].

**Scheme 10:**
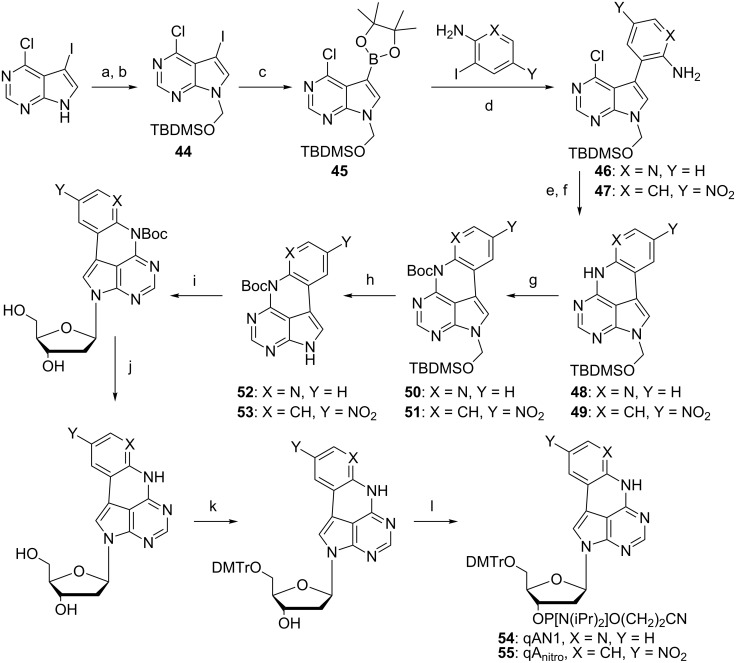
Synthesis of quadracyclic adenine base–base FRET pair. a) HCHO, NaOH, MeCN, H_2_O, 50 °C, 1 h; b) TBDMS-OTf, pyridine, 1 h, 0 °C to rt, 86% over two steps; c) HBPin, Pd(PPh_3_)_4_, Et_3_N, 1,4-dioxane, 24 h, 80 °C, 91%; d) PdCl_2_(PPh_3_)_2_, K_2_CO_3_, MeCN, H_2_O, 80 °C, 2 h, 86–95%; e) AcCl, pyridine, CH_2_Cl_2_, 3 h, rt; f) LiHMDS, THF, 100 °C, 2–6 h, 87–89%; g) Boc_2_O, DMAP, THF, 10 h, rt, 83–89%; h) ethane-1,2-diamine, TBAF, THF, 2 h, 0 °C to rt, 97–100%; i) NaH, Hoffer´s α-chloro sugar, MeCN, 2 h, 0 °C to rt, 55–69%; j) NaOMe, MeCN or MeOH, 1 h, 50 °C, 81–99%; k) DMTr-Cl, pyridine, 1.5 h, rt, 55–75%; l) 2-cyanoethyl-*N*,*N*-diisopropylchlorophosphoramidite, DIPEA, CH_2_Cl_2_, 2 h, rt, 87–90%.

Among the quadracyclic adenine analogues in those two studies we found qAN1 to be a promising candidate as a FRET donor due to its high quantum yield of 0.18 (vide infra) [55]. To develop an adenine acceptor for qAN1, a similar approach as for the tricyclic cytosines was performed, i.e., the introduction of a nitro functionality in the outer ring of qA. In an extensive investigation qA_nitro_ was synthesized and characterized and we found it, indeed, to be a suitable FRET acceptor for qAN1 (vide infra). The full synthesis scheme and characterization of this adenine–adenine analogue FRET pair was recently published by our group [25]. The synthetic approach was to first construct a common intermediate that could be used for various Suzuki-coupling partners similar to what we previously reported [55], by first protecting 6-chloro-7-iodo-7-deazapurine with *tert*-butyldimethylsilyloxymethyl (TBDMSOM) in 86% yield over two steps (**44**, Scheme 10). A Miyaura-type borylation afforded the common intermediate **45** in 91% yield and Suzuki coupling was then achieved efficiently for both 2-amino-3-iodopyridine as well as 2-iodo-4-nitroaniline in (**46**) 95% and (**47**) 86% yield, respectively. The activation of the exocyclic amine was achieved by using AcCl which provided a more robust cyclization using LiHMDS than if activating the amine using TMS-Cl. This furnished compounds **48** and **49** in 89% and 87% yield, respectively, over two steps. The subsequent Boc protection gave compound **50** in 89% and compound **51** in 83% yield. The quantitative TBDMSOM deprotection set the stage for a glycosylation using Hoffer’s α-chloro sugar and compounds **52** and **53** provided the desired β-anomer after purification in 69% and 55% yield, respectively. Global deprotection using sodium methoxide followed by standard DMTr-protection and phosphitylation provides the activated monomers for solid-phase synthesis [25]. The overall yield of qAN1 and qA_nitro_ phosphoramidite was 19% and 14%, respectively, which is significantly higher than our previous synthesis of qA (6% overall yield) starting from 6-chloro-7-iodo-7-deazapurine (Scheme 10).

### Photophysical properties of tricyclic cytosine analogues in nucleic acids

**Figure 3:**
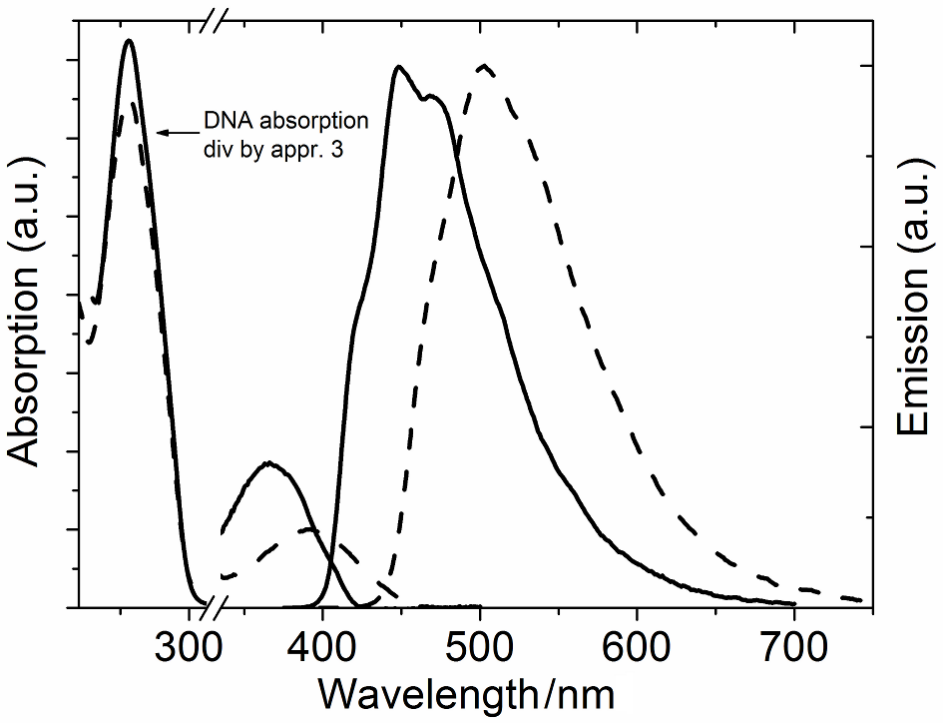
Absorption and emission of tC (dashed line) and tC^O^ (solid line) in dsDNA. The absorption below 300 nm is divided by three to emphasize the absorption spectral features of the lowest energy absorption bands of tC and tC^O^.

The tricyclic cytosine base analogues 1,3-diaza-2-oxophenothiazine (tC), and its oxo homologue, 1,3-diaza-2-oxophenoxazine (tC^O^) (Scheme 1 and Scheme 2) are both excellent fluorescent base analogues as well as donors for base–base FRET in nucleic acids [14,29–31,36]. Extensive evidences that both these base analogues mimic the behavior of natural cytosine have been found using UV–vis [30] and NMR spectroscopy [36], e.g., exchanging cytosine for one of them results in a virtually unperturbed B-form DNA helix. Importantly and as the first fluorescent base analogue with such properties, tC shows high and stable quantum yields (around 20%) both in monomeric form, in single- as well as in double-stranded DNA [29–30]. The quantum yield of tC^O^ in different environments is even higher than those of tC [31]. While slightly dependent on the neighboring base environment they are still very stable compared to other common fluorescent base analogues [15–16]. The absorption maxima of tC and tC^O^ in DNA are found at approximately 395 and 365 nm (Figure 3) [30–31], respectively, and, thus, are well separated from the absorption of the natural nucleobases. The emission of tC and tC^O^ in duplex DNA display large Stokes shifts, cover a broad wavelength region and the maxima are found at 505 and 450 nm (Figure 3), respectively [30–31]. Their spectral envelopes, which are an important factor for the overlap integral in FRET, are robust to changes in the local environment.

The fluorescence decays of tC are all monoexponential in single- as well as in double-stranded DNA resulting in a single lifetime of 5–7 ns depending on the sequence surroundings [30]. For tC^O^ single-stranded surroundings generally result in biexponential decays, whereas duplex surroundings, as in the case for tC, result in single fluorescence lifetimes (3–5 ns) [31]. High and stable quantum yields and single lifetimes in duplexes along with firm stacking are properties that make tC and tC^O^ excellent FRET donors. In order to make evaluation of FRET data more exact, through a high precision in the orientation factor (κ^2^), we have also determined the direction of the transition dipole moments of tC and tC^O^ (35° and 33° clockwise from the molecular long-axis as represented in Scheme 1 and Scheme 2, respectively) [31,35]. To complete the first base–base FRET pair there was a need for a FRET acceptor that could match tC and/or tC^O^. To this end we developed the nitro-version of tC, 7-nitro-1,3-diaza-2-oxophenothiazine (tC_nitro_) (Scheme 3) [14,38,45]. From UV–vis spectroscopy we showed that it, as do tC and tC^O^, forms stable, B-form duplexes and stacks firmly inside the DNA. It is a non-emissive chromophore that has an absorption maximum around 440 nm which overlaps well with the emission of both tC and tC^O^, thus, making it a potential FRET acceptor for both of them [14]. The best spectral overlap is found between the emission of tC^O^ and the absorption of tC_nitro_ giving a Förster radius (*R*_0_) of 27 Å using an isotropic orientation factor, κ^2^ = 2/3 [14]. Finally, for a high precision in orientation factor, i.e., to enable detailed structure investigations, we determined the direction of the lowest energy transition dipole moment of tC_nitro_ to be 25° in the opposite direction compared to tC and tC^O^ (i.e., pointing towards the nitro group) [14].

As was mentioned in the synthesis part above, recently we also have developed tC^O^ as an internal fluorophore for RNA systems [50]. The incorporation into RNA oligonucleotides and hybridization with a complementary strand results in normal A-form RNA duplexes. Moreover, the useful absorptive and emissive spectral properties of tC^O^ in DNA are retained in RNA. However, fluorescence decay data for tC^O^ in RNA suggests a less rigidly stacked conformation in RNA and two lifetimes are normally needed to achieve a good fit of the decays. With virtually stable quantum yields of 20–25% inside duplex RNA, tC^O^ is the brightest internal RNA fluorophore reported to date and, thus, a promising fluorescence reporter and/or FRET donor also in RNA systems [50].

### Photophysical properties of quadracyclic adenine analogues in nucleic acids

Extending the repertoire of base–base FRET pairs to other nucleobases would provide researchers the opportunity of replacing any sequence position in a nucleic acid with a base analogue FRET donor or acceptor. This motivated us to venture into the development of adenine analogues. Quadracyclic adenine (qA) [51], the emission of which was first reported by our group, was our initial adenine analogue candidate [53]. It stabilizes the native B-form DNA and is selective for base pairing with thymine. The emissive properties are decent both for the monomer (Φ_f_ = 6.8%) and inside DNA even though the quantum yield is quenched in the latter case. However, the average brightness in duplex DNA is still higher than that of 2-aminopurine and together with the excellent base-paring properties it is still a highly useful, environment-sensitive fluorescent-base analogue [53].

**Figure 4:**
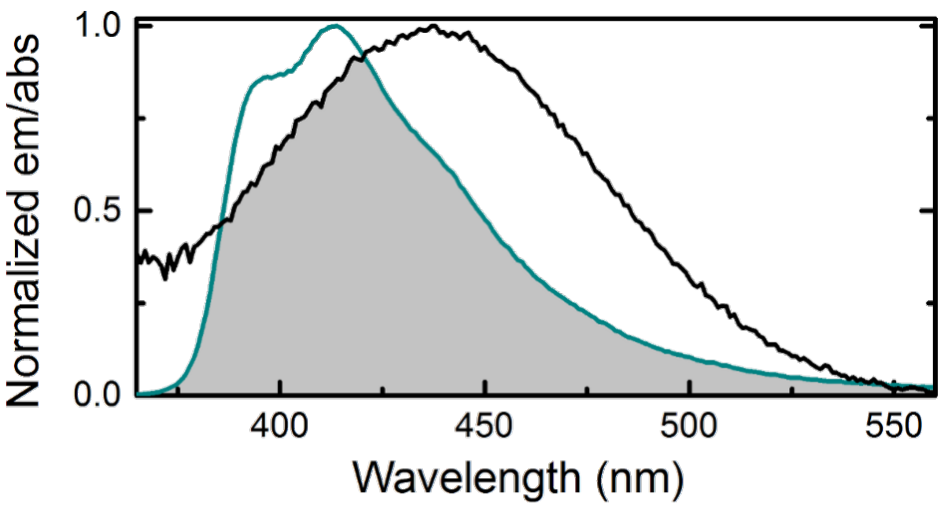
Spectral overlap between the emission of qAN1 (cyan) and the absorption of qA_nitro_ (black) in dsDNA. The shaded region constitutes the overlap integral (*J* integral).

Despite its excellent base-analogue properties, the low quantum yield of qA inside DNA disqualifies it for use as a base–base FRET donor. In order to maintain the base-analogue properties and achieve improved photophysical properties, we used quantum chemistry-supported design and developed a series of four, second generation, quadracyclic adenine analogues, qAN1–qAN4 (Scheme 9 and Scheme 10) [55–56]. As monomers, these compounds show significantly improved fluorescence properties. Importantly, one of the derivatives, qAN1, showed a high quantum yield in water (18%) that was not excessively influenced by varying the solvent, indicating that qAN1 is not highly sensitive to the direct surroundings [55]. Once incorporated into DNA strands, qAN1 specifically base-pairs to the complementary base, thymine, and allows formation of stable B-form DNA [25]. Moreover, the quantum yields inside DNA are significantly increased compared to those of qA. However, the quantum yields of qAN1 are slightly sensitive to the directly flanking bases with an average quantum yield of 6% in dsDNA [25]. The wavelength of the emission maximum found around 415 nm (Figure 4) in dsDNA is insensitive to the neighboring bases and the spectrum is more structured compared to the spectrum of monomeric qAN1, implying a firm stacking inside DNA [25]. The fluorescence lifetimes of qAN1 inside dsDNA show biexponential decays (average amplitude-weighted lifetimes ranging from 0.8 to 3.3 ns) for a majority of investigated sequences as compared to triexponential decays in ssDNA [25]. Overall, with a brightness (Φ_f_·ε = 510 M^−1^cm^−1^) inside DNA which is 29-times higher than for qA, specificity towards T and a firm stacking inside B-form DNA, qAN1 represents an excellent base–base FRET-donor candidate. To complete the base–base FRET pair the acceptor qA_nitro_ (Scheme 10) was designed and synthesized [25]. Spectroscopy-based investigations of the base analogue properties of qA_nitro_ inside DNA suggest that this derivative of qA is an excellent A-analogue just like qAN1. The lowest absorption maximum for qA_nitro_ in DNA is located at 435 nm (Figure 4) with a molar absorptivity of 5400 M^−1^ cm^−1^. As in the case of tC^O^ and tC_nitro_ there is an excellent spectral overlap between the emission of qAN1 and the absorption of qA_nitro_ (Figure 4) resulting in a Förster radius (using an orientation factor κ^2^ = 2/3) of 22 Å. This suggested that qAN1 and qA_nitro_ would constitute a good base–base FRET pair [25].

### Fluorescent base analogue FRET pairs inside DNA

**Figure 5:**
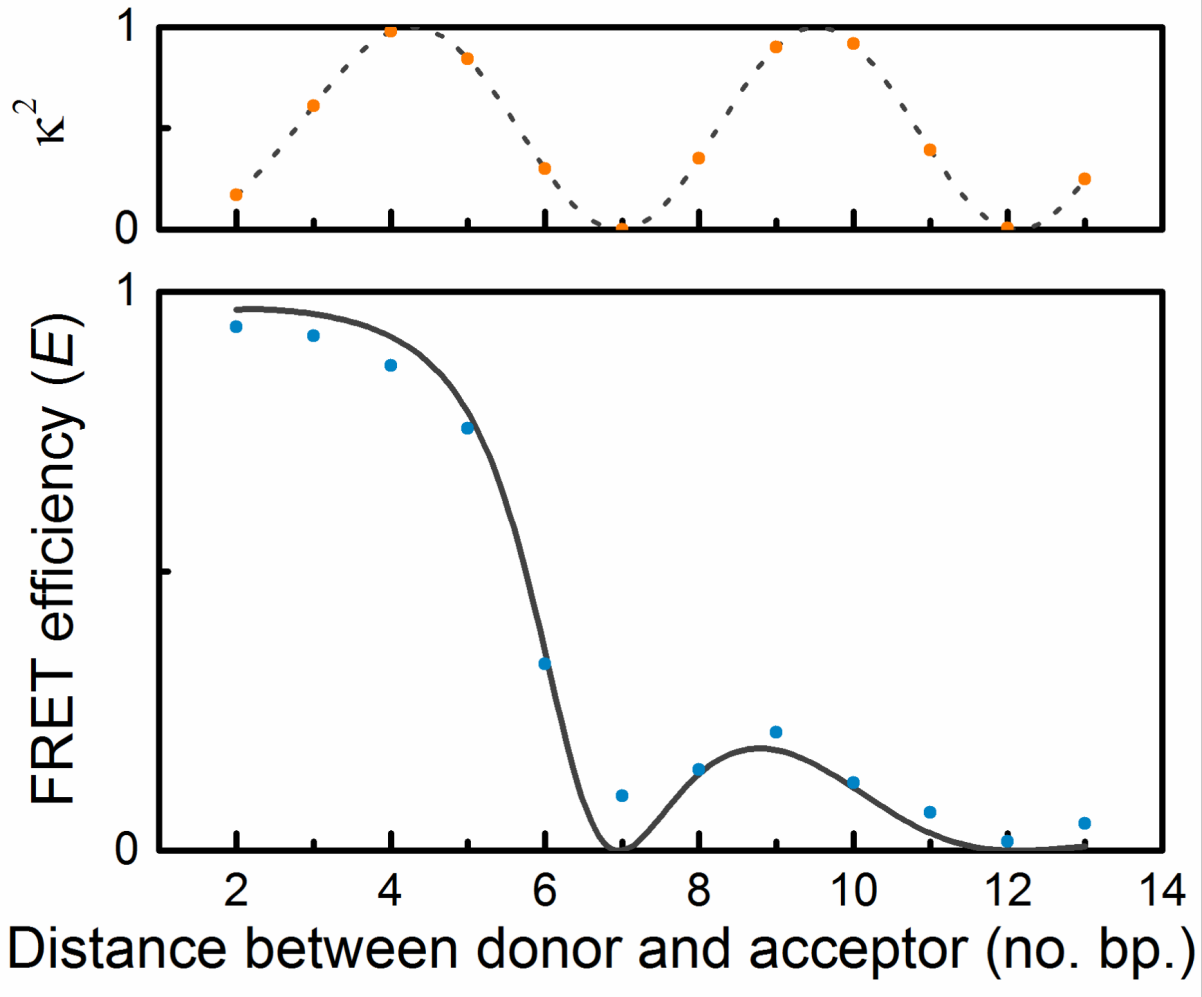
Example of typical FRET efficiency as a function of number of base pairs separating the donor and acceptor (data, blue dots, is an average of steady-state and time-resolved measurements of the FRET pair qAN1 and qA_nitro_). The line shows a curve fitted to the data based on FRET theory. The top graph shows how the orientation factor, κ^2^, varies with number of base pairs separating the donor and acceptor.

When using FRET theory on fluorophores/chromophores that replace nucleobases of a normal but static DNA, estimated energy-transfer efficiencies can be simulated using the structure parameters of the B-form duplex together with photophysical parameters of the fluorophores/chromophores. In this way we used the photophysical parameters we already had obtained for our two FRET pairs, tC^O^–tC_nitro_ and qAN1–qA_nitro_, to design the best combination of donor and complementary acceptor-containing DNA oligonucleotides [14,25]. We found that eight DNA strands were sufficient: three donor (tC^O^/qAN1) strands, four acceptor (tC_nitro_/qA_nitro_) strands and one unmodified strand serving as the complementary strand in donor-only reference samples. Combining these strands in an optimal way we covered distances of 2–13 bases separating the donor and the acceptor. For each base separation the FRET efficiency was investigated both by steady-state and time-resolved emission measurements. The results of those show an excellent resemblance with our predicted values for a nucleobase FRET pair situated inside a static DNA: an overall sharp (*R*^6^) decrease in the FRET efficiency with increasing numbers of bases separating the donor and the acceptor with an overlaid sinusoidal curvature as a consequence of the effect of the helical nature of B-form DNA on the orientation factor, κ^2^ (Figure 5) [14,25]. Both, the measured sets of FRET efficiencies, the one for tC^O^–tC_nitro_ as well as the one for qAN1–qA_nitro_, were fitted to an averaged, static B-form DNA model using an in-house built MATLAB script. The best fits agree excellently with the measured data and suggest that our two FRET pairs are indeed rigidly stacked inside DNA and serve as excellent distance and orientation dependent FRET probes (Figure 5) [14,25]. In the fit we used the associated phase angle (angle between the transition dipole moments of the donor and the acceptor) and the spectral overlap (*J*_DA_) as the fitting parameters. The phase angles for the tC^O^–tC_nitro_ and the qAN1–qA_nitro_ FRET pairs were 67° and 33°, respectively, that are in good agreement with the experimentally determined one for the cytosine analogue FRET pair (58°) and the TDDFT-estimated one for the adenine analogue FRET pair (41°) [14,25]. Also the spectral overlap integrals show high similarity to the values resulting from the best fit. Taken together these two FRET pairs comprise excellent tools to study detailed structure, dynamics and conformational changes of DNA. An additional advantage with our cytosine and adenine analogue FRET pairs is that they, as a result of their spectral features, can be combined with each other, i.e., qA_nitro_ can replace tC_nitro_ as an acceptor of tC^O^, and, similarly, tC_nitro_ can replace qA_nitro_ as an acceptor of qAN1 [25]. A useful advantage of this is that we can now perform base–base FRET between any sequence positions inside duplex DNA.

Recently Sugiyama and co-workers reported a nucleobase-analogue FRET pair that consists of the 2-aminothieno[3,4-*d*]pyrimidine G-mimic deoxyribonucleoside (^th^dG) (see Figure 2) [23], developed by the Tor lab, as an energy donor and 1,3-diaza-2-oxophenothiazine (tC), developed by our lab, as an energy acceptor [57]. This G–C analogue FRET pair also displays the general characteristics of an energy efficiency curve of base–base FRET and is able to emit cyan-green light from its acceptor molecule tC. The authors used this FRET pair to study a change from B-to-Z-form DNA using the color and intensity change of the combined donor and acceptor emission [57].

### FRETmatrix

To enable detailed studies using our FRET pairs we have developed the freely available software FRETmatrix [58]. It consists of two parts, one that predicts FRET efficiencies from structural input and one that can calculate the most probable structure using measured FRET efficiencies as input.

The first part is useful in the design of a study, as it can predict the change in FRET efficiency between two base analogues upon a structural change of the DNA (for example caused by protein binding). This allows the user to make informed choices of where in a DNA duplex to incorporate the modified bases to get useful FRET-change responses. The second, more powerful part provides structural information based on the FRET efficiencies measured between base analogues positioned on opposite sides of a constraint site. The constraint site can, for example, be a protein-induced kink in the DNA. The software needs the DNA sequence together with photophysical data of the FRET pair and measured FRET efficiencies as input. Then, assuming the rest of the DNA is unchanged, the geometrical parameters of the constraint site can be obtained. This is possible since the base analogues are rigidly positioned inside the DNA (Figure 1c) and the FRET efficiencies depend on the relative distance and angle between them (see Equations 1–3). FRETmatrix, in this way provides a convenient possibility to study structural changes of nucleic acids in solution using only emission measurements [58]. For example, in a small demonstration study we have shown that the method can be used to resolve the structure of a 3A (3 adenine) bulge [58]. The same bulge has been studied by other groups as well, using different techniques and with similar results [59–61]. An elegant and groundbreaking way to study and use detailed FRET has also been reported by Seidel et al. By assuring that the external dyes in use are truly free to rotate (see Figure 1b; isotropic orientation) an orientation factor, κ^2^, of 2/3 can be assumed. In combination with advanced computer modeling, high accuracy structural parameters can thus be resolved from FRET measurements [62–63]. In conclusion, the FRETmatrix (base–base FRET) and Seidel’s methodology are in a way two extremes, firmly vs randomly oriented probes, both giving high control of the κ^2^ value which in turn facilitates high detail structure information determination.

### Studying nucleic acid conformation and conformational changes using base–base FRET

Many biologically important processes such as binding of transcription factors to DNA, polymerase–DNA interactions during replication, gene regulatory systems and structure variation due to changes in conditions (e.g., B-to-Z-form DNA), generally involve conformational changes where base–base FRET can be used with an advantage. The possibility to monitor both, distance and orientation of these conformational changes and inherent dynamics of the systems in real time increases the level of detail accessible in the FRET investigation. Over the less than ten years they have been available, nucleobase analogue FRET pairs have been able to monitor several important processes including transcription and DNA repair. Here we give a short summary of a number of those applications.

#### Higher detail structure information investigations

DNA exists in a variety of conformations depending on conditions. Z-DNA, a GC-repeat rich, thermodynamically less preferred, left-handed helical conformation that is favored by cytosine methylation is known to form in vivo under negative supercoiling or high salt concentrations [64–68]. Circular dichroism is traditionally the predominant method to investigate Z-DNA and to monitor conformational changes from B-to-Z-form DNA [68–72]. However, the development of nucleobase analogue-based FRET provided an opportunity to sense the significant orientational and distance changes for the B-to-Z-transition in real time using significantly smaller sample amounts. Therefore, we set out to use the tC^O^–tC_nitro_ FRET pair to develop new methodologies to investigate Z-form DNA [73]. Two different DNA constructs were selected: one of them containing a (GC)_7_ and the other a (GC)_5_. The former is a hairpin which is designed to be able to transform completely into Z-form DNA at high salt concentrations and the latter is able to form a B–Z DNA junction under similar, high salt conditions. The tC^O^–tC_nitro_ FRET pair was incorporated at three different base separations (4, 6, and 8 bases between donor and acceptor, respectively). The results show significant changes in the FRET efficiencies upon B-to-Z-DNA transition (e.g., from 35 to 8%) that can, not only, be used to monitor the presence of Z-form DNA but also to determine the rate constants for these transitions [73]. We showed in this investigation that the FRET-based method to study Z-form DNA reduces the amount of sample needed by almost three orders of magnitude compared to the most commonly used CD methodology [73].

**Figure 6:**
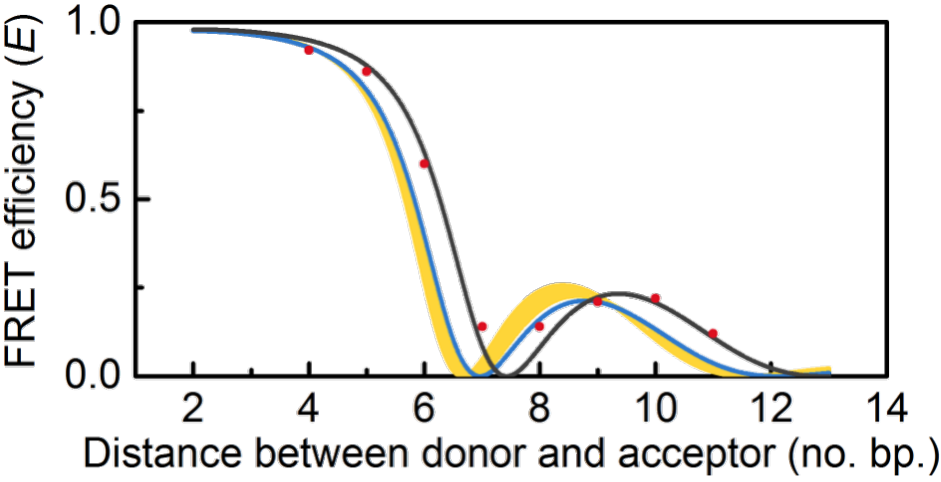
FRET efficiency as a function of number of base pairs separating the donor (qAN1) and acceptor (qA_nitro_). Red dots mark the measured FRET efficiency with netropsin bound. The black line shows the best fit to the data based on FRET theory. The blue line shows the curve for B DNA. The yellow area depicts the range possible if each netropsin molecule overwinds the DNA as stated in previous literature.

Recently we used our adenine analogue FRET pair, qAN1–qA_nitro_, to study the conformational change of B-form DNA upon interaction with the established minor groove binder netropsin [25]. Netropsin is an archetypal minor groove ligand that binds short (4–5 bp) AT-rich sequences [74–76]. In our investigation we first measured the FRET efficiencies, using both steady-state and time-resolved emission, between qAN1 and qA_nitro_ separated by 2–13 bp in a B-form DNA. Thereafter, we added netropsin until site saturation and again measured the FRET efficiencies (Figure 6), now for base separations of 4–11 bp [25].

Upon netropsin binding the measured base–base FRET efficiencies change significantly in their orientational component (extreme values are shifted to larger base separations) and also slightly in their distance component (shows up as a higher amplitude for the maximum around 9–10 bp). Fitting these FRET data (Figure 6) points to the best possible DNA helical twist and rise values results in a decrease in twist and rise by 2° and 0.25 Å, respectively [25]. This is in contrast to previously reported values showing slight increases in helical twist and rise measured, for example, by sedimentation [77], gel electrophoresis [78–79], X-ray crystallography [74] and magnetic tweezers [75]. In our investigation we modeled the general appearance of a FRET curve resulting from such, small, helical twist and rise increases (yellow area in Figure 6) and in this way were able to establish that our data unambiguously show decreases in twist and rise [25]. One important difference with our system compared to most previous studies is the fact that we use short DNAs that easily can relax the strain induced by the netropsin binding. Our netropsin study shows the general strength and potential of base–base FRET to investigate even very small changes in distance and orientation and the finding warrants further studies of this structural change using a larger set of DNA sequences containing netropsin binding sites.

#### Qualitative base–base FRET to investigate vital cellular processes

As clearly shown above, base–base FRET is a powerful method to obtain structure information with high structure detail. However, one of its obvious and even more straightforward applications is merely for monitoring whether a certain process involves a conformational change or not. A few examples of such applications are described below.

In a collaborative investigation with Falkenberg and Gustafsson, we investigated the role of the transcription factor A (TFAM) in the mitochondrial transcription machinery [80–81]. The investigation, also involving an extensive use of gel electrophoresis studies, shows that TFAM, in contrast to previous reports, indeed is a core component of the machinery. In the study our FRET pair tC^O^–tC_nitro_ was site-specifically incorporated in various positions close to the HSP1 transcription initiation site. The results suggest that when TFAM binds to the DNA, it causes significant structural changes [80]. These changes are clearly visible in the tC^O^–tC_nitro_ FRET data that also indicate that the conformational changes could be consistent with DNA breathing. Moreover, the data demonstrated that the structural changes upon binding of TFAM near the transcription initiation site are the result of sequence-independent binding to DNA. The investigation establishes the potential of using base–base FRET for studying nucleic acid conformations in vital cellular processes without perturbing the system under study.

In another report using the tC^O^–tC_nitro_ FRET pair as a probe of protein interaction, Ansari et al. investigated the DNA damage repair system [82]. Here the FRET pair is used to better understand the conformational dynamics along the DNA-lesion recognition trajectory. The tC^O^–tC_nitro_ FRET pair was incorporated on both sides of mismatched regions in a DNA to report on conformational changes upon DNA repair protein Rad4 interaction. The FRET data obtained support a model in which Rad4 binds to the mismatched part causing a “twist-open” mechanism and demonstrates the potential of base–base FRET in short time-scale kinetics investigations [82].

## Conclusion

Base–base FRET has a great potential as a detailed structure and dynamics tool in biomolecular sciences. It serves as an interesting complement to FRET pairs based on external fluorophores enabling higher structure resolution and monitoring of a different distance range with high accuracy. With the recent advent of new base–base FRET pairs, the coming years offer great prospects for increased use of such methodology. The combination of base–base FRET and single-molecule-based FRET on nucleic acids with external probes as developed by Seidel et al. [4] comprise a highly interesting opportunity to investigate structure and dynamics of nucleic acid containing systems. Recent progress in the field of fluorescent base analogues also starts to close the gap in brightness to external fluorophores like Cy-, Alexa- and ATTO-dyes and the development of a base analogue with properties that are satisfactory for single molecule use would open up completely new possibilities to study the detailed structure, dynamics and conformational changes of one of the key players in life: nucleic acids.
